# Pulmonary talcosis due to aspiration

**DOI:** 10.36416/1806-3756/e20240401

**Published:** 2025-03-18

**Authors:** Edson Marchiori, Bruno Hochhegger, Gláucia Zanetti

**Affiliations:** 1. Universidade Federal do Rio de Janeiro, Rio de Janeiro (RJ) Brasil.; 2. University of Florida, Gainesville (FL) USA.

**Figure 1:**
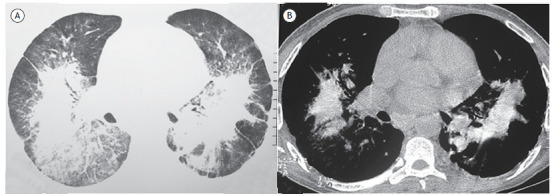
CT scans of the lower lobes with lung window setting (in A) showing bilateral conglomerate masses, ground-glass opacities, areas of emphysema, and dense streaks in the periphery. In B (mediastinal window setting), areas of increased attenuation within the conglomerate masses are revealed, compatible with talc deposition.

A 41-year-old man with dry cough and progressive dyspnea on moderate/high exertion was admitted to our service. He worked for 8 years in a cosmetics industry. A chest CT scan showed bilateral conglomerate masses (Figure 1). Tomographic findings associated with the occupational history allowed the diagnosis of pulmonary talcosis.

Conglomerate masses can occur in four basic pulmonary conditions: silicosis, coal workers’ pneumoconiosis, sarcoidosis, and talcosis. Although the identification of conglomerate masses on chest CT restricts the diagnostic possibilities to these four diseases, clinical and occupational history, both current and past, is essential for final diagnosis.

Talcosis is an uncommon pneumoconiosis, related to the aspiration or injection of talc (magnesium silicate). Patients may be asymptomatic or present with a severe disease course. Symptomatic patients usually present with nonspecific complaints, including progressive dyspnea on exertion and cough.

Late complications include chronic respiratory failure, emphysema, pulmonary arterial hypertension, and *cor pulmonale*. Two distinct forms of lung disease caused by talc have been defined. One is associated with aspiration of the product, and one results from intravenous administration of talc, seen in drug users. Talc is a mineral widely used in various industries. Inhalation of a large amount of talc can occur during its extraction from mines, separation, milling, packaging, loading, and transportation. There have also been reports of talcosis in workers exposed to talcum powder in secondary industries such as rubber, paper, textiles, leather, ceramics, pharmaceuticals, cosmetics, insecticides, and herbicide manufacturers, as well as in soapstone workers. In addition to the possible occupational history, the possibility of the patient being a drug addict should be carefully evaluated, especially those who intravenously inject oral substances.

On CT, findings of small centrilobular nodules associated with heterogeneous conglomerate masses containing amorphous areas of high attenuation within them, determined by talc deposition, with or without panlobular emphysema in the lower lobes, are highly suggestive of pulmonary talcosis. On CT, the main difference between inhaled and intravenous forms is the possibility of emphysema developing in the latter. The histopathological feature of talc pneumoconiosis is the presence of birefringent, needle-shaped talc particles seen within giant cells and in areas of pulmonary fibrosis using polarized light.

Our patient also underwent BAL, which showed birefringent particles, confirming the diagnosis of pulmonary talcosis.
